# Different effects of two mutations on the infectivity of Ebola virus glycoprotein in nine mammalian species

**DOI:** 10.1099/jgv.0.000999

**Published:** 2018-01-04

**Authors:** Yohei Kurosaki, Mahoko Takahashi Ueda, Yusuke Nakano, Jiro Yasuda, Yoshio Koyanagi, Kei Sato, So Nakagawa

**Affiliations:** ^1^​ Department of Emerging Infectious Diseases, Institute of Tropical Medicine (NEKKEN), Nagasaki University, 1-12-4 Sakamoto, Nagasaki 852-8523, Japan; ^2^​ Micro/Nano Technology Center, Tokai University, 411 Kitakaname, Hiratsuka, Kanagawa 259-1292, Japan; ^3^​ Laboratory of Systems Virology, Department of Biosystems Science, Institute for Frontier Life and Medical Sciences, Kyoto University, 53 Shogoinkawahara-cho, Sakyo-ku, Kyoto 606-8507, Japan; ^4^​ Graduate School of Biomedical Sciences and Program for Nurturing Global Leaders in Tropical and Emerging Communicable Diseases, Nagasaki University, 1-12-4 Sakamoto, Nagasaki 852-8523, Japan; ^5^​ CREST, Japan Science and Technology Agency, Saitama 322-0012, Japan; ^6^​ Department of Molecular Life Science, Tokai University School of Medicine, 143 Shimokasuya, Isehara, Kanagawa 259-1193, Japan

**Keywords:** Ebola virus, Reston virus, glycoprotein viral infectivity, Niemann-Pick C1, virus-host interaction

## Abstract

Ebola virus (EBOV), which belongs to the genus *Ebolavirus*, causes a severe and often fatal infection in primates, including humans, whereas Reston virus (RESTV) only causes lethal disease in non-human primates. Two amino acids (aa) at positions 82 and 544 of the EBOV glycoprotein (GP) are involved in determining viral infectivity. However, it remains unclear how these two aa residues affect the infectivity of *Ebolavirus* species in various hosts. Here we performed viral pseudotyping experiments with EBOV and RESTV GP derivatives in 10 cell lines from 9 mammalian species. We demonstrated that isoleucine at position 544/545 increases viral infectivity in all host species, whereas valine at position 82/83 modulates viral infectivity, depending on the viral and host species. Structural modelling suggested that the former residue affects viral fusion, whereas the latter residue influences the interaction with the viral entry receptor, Niemann–Pick C1.

The genus *Ebolavirus* belongs to the family *Filoviridae* and currently contains five species: the *Zaire*, *Sudan*, *Bundibugyo*, *Taï Forest* and *Reston ebolaviruses* [1]. Infection with Ebola virus (EBOV) of the *Zaire ebolavirus* species causes severe haemorrhagic fever in primates, including humans, with a high mortality rate [2]. Unlike viruses from the other *Ebolavirus* species, Reston virus (RESTV) infection (species *Reston ebolavirus*) is relatively apathogenic in humans, but causes lethal illness in non-human primates [3]. Although molecular and serological evidence of EBOV and RESTV infection in fruit bats has been reported, the natural reservoirs of these viruses have not yet been determined [4–6].

EBOV glycoprotein (GP) is a surface viral protein and is responsible for viral receptor binding and cell entry. Recent studies of this GP have shown that two EBOV GP mutations, alanine to valine at position 82 (A82V) and threonine to isoleucine at position 544 (T544I), may cause an increase in viral infectivity in humans [7–12]. Although Ruedas *et al.* recently reported that a T544I mutation in EBOV GP may be selected during tissue culture propagation [13], EBOV GP mutants with V82 and I544 reduce the stability of the prefusion conformation of EBOV GP. The A82V mutation promotes the conformational changes induced by Niemann–Pick C1 (NPC1) binding, and the T544I mutation mediates membrane fusion and infection [11]. The frequency of these two alleles (A/V at 82 and T/I at 544) differs between *Ebolavirus* species. In particular, RESTV GP isolated from clinical specimens of pig possesses A and I at positions 83 and 545, corresponding to positions 82 and 544 of EBOV GP, respectively (Fig. 1a). Additionally, since the pathogenicity of RESTV and EBOV in humans differs, it is plausible to assume that the importance of these two residues for viral infection differs between viral and/or host species.

**Fig. 1.**
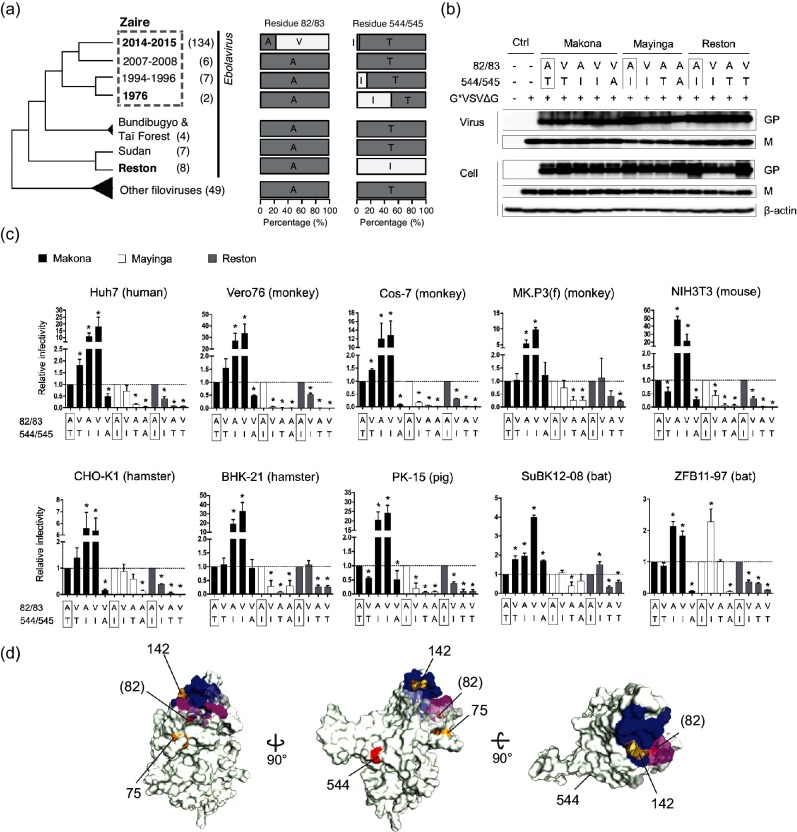
Infectivity of pseudotyped vesicular stomatitis viruses with Ebola virus or Reston virus glycoprotein in mammalian cell lines. (a) Allele frequencies of glycoproteins (GPs) of Ebola virus (EBOV) or Reston virus (RESTV) species at positions 82/83 and 544/545 with filovirus phylogeny. Species names in bold are the species used in the pseudotype assays. EBOV is further divided into four variants by the outbreak year. The years 2014–2015 and 1976 represent the Makona and Mayinga isolates, respectively. Bundibugyo virus contains a GP sequence that is closest to that of the species Taï Forest. The numbers in parentheses next to the species names indicate the numbers of the 147 representative GP sequences obtained from [7]. GP sequences from 48 Marburg viruses and one Cueva virus were used as outgroups. (b) GP and vesicular stomatitis virus (VSV)-M incorporated into virions or expressed in the virus-producing cells were detected by immunoblotting using anti-GP or anti-VSV-M monoclonal antibodies. Boxes indicate amino acid (aa) residues in wild-type GP from Makona EBOV (*H. sapiens*/GIN/2014/Makona/Gueguedou-C15; GenBank accession number KJ660346.2), Mayinga EBOV (*H. sapiens-tc*/COD/1976/Yambuku-Mayinga; GenBank accession number AF086833.2) and RESTV (*M. fascicularis-tc*/USA/1989/Philippines89-Pennsylvania; GenBank accession number NC_004161.1) at the indicated positions. The cells and culture supernatants transfected with empty vector (pCAGGS) and G*VSVΔG- or mock-infected cells were used as controls. β-actin was detected as a loading control. (c) The infectivity of each virus was quantified by measuring luciferase activity in the infected cells at 24 h post-infection using the Steady-Glo luciferase assay system (Promega Corp., Madison, WI, USA) and a TriStar LB 941 microplate reader (Berthord Japan K.K., Tokyo, Japan). The relative infectivity of the wild-type or mutant Makona EBOV (black), Mayinga EBOV (white) and RESTV (grey) was determined by setting the value of wild-type EBOV/RESTV to 1.0. The aa residues of wild-type viruses at each position are boxed. The data shown represent the mean and standard error of three independent experiments. Statistical analysis was performed using Student’s *t*-test (*, *P*<0.05) in Prism 6 (GraphPad Software Inc., La Jolla, CA, USA). (d) The molecular surface of EBOV GP is shown. GP forms a trimeric spike, but only a monomer is shown for clarity. The aa positions are based on the crystal structure of 5F1B [21]. The NPC1 receptor-binding residues around position 82 and the substitution residues found in RESTV (142/143 and 75/76) are highlighted in magenta and orange, respectively. The blue surface also represents receptor-binding residues that are not located around residue 82. Blue indicates receptor-binding domain residues at positions 111–114, 118, 141–147, 152 and 170.

To address this hypothesis, we conducted experiments with *in vitro* cell culture systems using the pseudotyped vesicular stomatitis virus (VSV) bearing GP derivatives from RESTV and two EBOV isolates (Makona and Mayinga) in various mammalian cell lines (summarized in Table 1). The GP-containing pseudotyped VSV was prepared in 293 T cells using recombinant VSV in which the *luciferase* gene was substituted for the *VSV-G* gene (G*VSVΔG) and RESTV or EBOV GP-expressing plasmids, as previously described [7, 14]. To confirm the amount of virus used in each experiment, viral proteins in the pseudotyped viruses precipitated by ultracentrifugation and virus-producing cells were detected by Western blotting using monoclonal antibodies for the matrix protein VSV-M (23H12; Kerafast, Boston, MA, USA) and GP [15]. Each virion bearing the reported residue (termed ‘wild-type’ here) or mutant GP had a similar amount of M and GP in respective isolates despite the presence of GP mutations, indicating that consistent amounts of pseudotyped viruses were inoculated into the cells, and GPs were equally incorporated into each virion regardless of mutations at these two positions. The differences in the infectivity of each mutation were dependent on the functional alterations of each GP rather than being the result of protein concentration (Fig. 1b). The infectivity of each pseudotyped VSV bearing RESTV or EBOV GP was determined by luciferase activity in the infected cells at 24 h post-infection (Fig. S1, available in the online version of this article). The viral infectivity of single or double mutants at the two positions was determined relative to that of the wild-type form of each virus (Fig. 1c).

**Table 1.** Cells and GenBank accession IDs of Niemann–Pick C1 used in this study

Cell line	Common name	Scientific name	Tissue	GenBank accession ID of NPC1*
Huh7	Human	*Homo sapiens*	Liver	XP_005258334
Vero76	African green monkey	*Cercopithecus aethiops*	Kidney	XP_007972965
Cos-7	African green monkey	*Cercopithecus aethiops*	Kidney	XP_007972965
MK.P3(f)	Cynomolgus monkey	*Macaca fascicularis*	Kidney	XP_005587087
NIH3T3	Mouse	*Mus musculus*	Embryo	NP_705888
BHK-21	Syrian golden hamster	*Mesocricetus auratus*	Kidney	XP_012979687
CHO-K1	Chinese hamster	*Cricetulus griseus*	Ovary	NP_001233616
PK-15	Pig	*Sus scrofa*	Kidney	NP_999487
ZFB11-97 [22]	Gambian epauletted fruit bat	*Epomophorus gambianus*	Kidney	n.a.
SuBK12-08 [22]	Schreiber's bat	*Miniopterus schreibersii*	Kidney	n.a.

*NPC1, Niemann–Pick C1.

First, we assessed the impact of the residue at position 544/545 of EBOV/RESTV. Consistent with our recent report [7], the T544I mutation in Makona GP significantly increased viral infectivity (10.9-fold; *P*<0.05) in Huh7 cells (Fig. 1c), whereas the I544T mutation in Mayinga GP significantly decreased its infectivity (0.38-fold; *P*<0.05). The I545T mutation in RESTV GP decreased its infectivity in Huh7 cells (0.08-fold; *P*<0.05), and similar results were observed in all the mammalian cell lines examined in this study (Fig. 1c). These findings suggest that isoleucine at position 544/545 augments the infectivity of EBOV and RESTV, regardless of host species. Based on the crystal structure of EBOV GP, residue 544 comprises the hydrophobic surface of an internal fusion loop (IFL) that is critical for viral membrane fusion with late endosomes [16]. An alanine mutation at 544 (I544A) decreased the IFL activity, due to the spread of residues forming a hydrophobic surface. Consistent with a previous study [17], the mutation to alanine at 544 in both Makona (T544A) and Mayinga (I544A) decreased viral infectivity in mammalian cell lines (Fig. 1c). Since the interaction between this IFL and the endosomal membrane occurs independently of specific cellular proteins, it is possible to speculate that residue 544 contributes to viral fusion activity in the mammalian cell lines examined in this study.

In contrast, the effects of A82/83V on viral infectivity in human and other host species differed between *Ebolavirus* species (Fig. 1c). Previously, we used a pseudotyped virus assay in Huh7 cells to show that the A82V single mutation in Makona GP significantly increases viral infectivity (1.81-fold; *P*<0.05) [7]. The A82V mutation in Makona GP also increased viral infectivity in Cos-7 and SuBK12-08 cells (*P*<0.05). However, for the Makona GP A82V mutant, reduced infectivity was detected in NIH3T3 and PK-15 cells (*P*<0.05), and no statistically significant differences in infectivity were observed in the other six cell lines. Indeed, reduced infectivity of the A82V mutation in Mayinga GP was evident in half of the cell lines examined (Vero 76, Cos-7, NIH3T3, BHK-21 and PK-15 cells; *P*<0.05), although increased infectivity was observed in ZFB11-97 cells (*P*<0.05). These results suggest that the effect of the A82V mutation in EBOV GP on viral infectivity differs among both *Ebolavirus* and host species. We also found that the A83V single mutation in RESTV GP significantly decreased viral infectivity in 7 out of 10 cell lines (Huh7, Vero76, Cos-7, NIH3T3, CHO-K1, PK-15 and ZFB11-97 cells; *P*<0.05), whereas a significant increase was only observed in SuBK12-08 cells (*P*<0.05). In the remaining two cell lines (MK.P3 and BHK-21), there was no statistical difference in the entry efficiency between A83V mutations in RESTV. These results suggest that the A83V mutation in RESTV GP is detrimental to RESTV infection of some, but not all, cell types. Moreover, the increased infectivity generated by the A82V mutation in the human cell lines tested here may be a limited feature unique to Makona EBOV variants. Whereas alanine in this position is highly conserved in all isolates of RESTV or Mayinga EBOV, as well as in other filoviruses (Fig. 1a), valine is only beneficial in Makona GP for efficient infection in certain host species, including humans. As we reported previously, the original Makona isolates had A82 in their GP, but the A82V mutation was positively selected for and fixed during transmission in humans during the 2014–15 outbreak [7–9, 11, 12]. Our results suggest that Makona but not Mayinga or RESTV GP may have specifically acquired the A82V mutation that increases infectivity. We also found that the A82/83V mutation showed different effects on the infectivity in the two bat cell lines (SuBK12-08 and ZFB11-97) used in this study. The cell line SuBK12-08 specifically increased the susceptibility of the virus with A82V in Makona GP and A83V in RESTV GP, whereas the cell line ZFB11-97 increased susceptibility of the virus with A82V in Mayinga GP, suggesting that the effect of a mutation at position 82/83 for viral infectivity is complex (Fig. 1c). Fruit bats are strongly suspected to be a natural reservoir of Ebola viruses, in which new variants of the viruses may have emerged. Bat cells are more likely to accept GP mutations than other host species.

To better understand the impact of residue 82/83 in the EBOV/RESTV GPs, we compared GP amino acid (aa) sequences. When comparing EBOV GP sequences between Makona and Mayinga isolates, excluding residues 82 and 544, only one aa variation was found at residue 503 in the non-disordered GP structure. However, residue 503 is not involved in the receptor-binding domain (RBD) of NPC1 that is important for the entry of filoviruses into host cells [18–21]. The discrepancy in the infectivity between the two EBOV isolates may derive from the effect of different combinations of these aa on the infection process after the GP–NPC1 binding [9]. Next, we compared the aa sequences of the EBOV and RESTV GPs. EBOV and RESTV are phylogenetically distinct (Fig. 1a), and the aa identity of their GP is only ~58 %. Although the RBDs of the EBOV and RESTV GPs are almost identical, we detected differences in the residues at positions 75/76 and 142/143 between them (Fig. 1d). These aa differences may be associated with the different effects of the A82/83V mutation on their infectivity. Indeed, *in silico* structural analyses suggested that the RBD of RESTV GP has relatively low surface hydrophobicity and a high electrostatic potential compared with that of Mayinga EBOV, especially in residues around position 143 in RESTV GP (Figs S2–S4). The difference in these properties between the EBOV and RESTV GPs may affect their affinity for NPC1, which may change the effect of the A82/83V mutation in different host species. Further comparison of the surface electrostatic potential between wild-type and A83V mutant RESTV GPs indicated that the A83V mutation extends the non-charged surface around position 83 (Fig. S2). This may explain the difference in infectivity between wild-type and A83V mutant RESTV GPs.

For EBOV and RESTV species, we found that the same mutation had different effects on infectivity in host species depending on the aa combinations of the 82/83 and 544/545 residues. To understand the differences in the infectivity of EBOV/RESTV in host cells, we compared the aa sequences of loop2 and the surrounding regions of the C domain of NPC1 in the 10 mammalian cell lines examined in this study (Fig. 2a, b). In all of the species examined, aa substitutions were found in/around loop 2, many of which were located upstream of the C domain of NPC1, except for the residue at position 521 (Fig. 2a). Of the 14 mutations, 7 showed significant changes in mutation energy (Figs 2a, b and S5) when introduced into human NPC1. The results of simulations showed both stabilizing and destabilizing effects. The mutation sites were often found in areas with low hydrophobicity and high electrostatic potential (Fig. S5). Conversely, two mutations within the NPC1–GP interacting residues did not show significant changes in mutation energy, but instead, the hydrophobicity of the mutated residues was altered (Fig. 2a). This implied that these mutations affect the biochemical characteristics and structure around loop 2, changing the sensitivity and structure of the interaction surface by altering the NPC1–GP interaction.

**Fig. 2.**
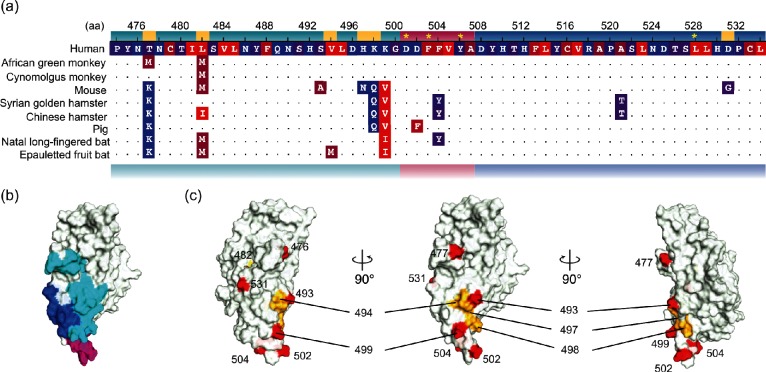
Amino acid variations and structures of the C domain of mammalian Niemann–Pick C1. (a) Amino acid (aa) alignments around loop2 of Niemann–Pick C1 (NPC1). The amino acid (aa) positions shown above are based on the human NPC1 structure of 5F1B [21]. Yellow asterisks on the human sequence indicate residues that interact with aa at positions 80 and 83 of *Zaire ebolavirus* glycoprotein. Dots represent residues in common with the human sequence. The aa are coloured according to the hydrophobicity from hydrophobic (red) to hydrophilic (blue). Loop2 and its upstream and downstream sequences are coloured in magenta, turquoise and blue, respectively. Yellow highlighted residues are those with a significant change in mutation energy between human wild-type and mutant forms generated by substitutions in non-human NPC1. Since no NPC1 sequences for the bat species used in our experiments are publicly available, different species in the same genera/subfamilies were used for the sequence comparison; *Miniopterus natalensis* and *Epomops buettikoferi* were used for *Miniopterus schreibersii* and *Epomophorus gambianus*, respectively [23]. (b) Schematic of the structure of the NPC1 C domain (5F1B [21]) at the molecular surface. The colours are the same as in panel (a) of this figure. (c) The locations of mutations are shown (red). The locations of residues with a significant change in mutation energy are also highlighted using the same colours as in panel (a).

We showed that the residues at positions 82/83 and 544/545 of the Makona EBOV, Mayinga EBOV and RESTV GPs have a pronounced effect on viral infectivity in the 10 mammalian cell lines. Our findings indicate that these two residues commonly affect the efficiency of infection of EBOV and RESTV, although the effects of the mutations vary greatly. The effect of the V82/83 residue on viral infectivity differs among both *Ebolavirus* and host species, whereas the residue I544/545 of EBOV and RESTV GPs increases viral infectivity in various host species. The molecular functions of both residues at positions 82/83 and 544/545 of EBOV GPs in natural hosts remain unclear. Further analyses are needed to understand the functions of these key residues for the infection of *Ebolavirus* species in animals and humans.

## Supplementary Data

13639 Supplementary File 1
