# Supplementary material for: Different effects of two mutations on the infectivity of Ebola virus glycoprotein in nine mammalian species
Source: J Gen Virol. 2018 Jan 4;99(2):181–6. doi: 10.1099/jgv.0.000999 (PMC5882082; doi:10.1099/jgv.0.000999)

Supplementary information

Fig. S1. Efficiency of gene transduction into various mammalian cell lines by EBOV or RESTV GP.

The infectivity of each pseudotyped VSV bearing EBOV or RESTV GP in the 10 mammalian cell lines was determined as relative light unit (RLU) as described in Fig.1. The amino acid residues of wild-type viruses at each position are boxed. The data shown represent mean and standard deviation of three independent experiments. Pseudotyped viruses with or without VSV G (ΔG) serve as positive or negative controls, respectively.

Fig. S2. Hydrophobicity of the glycoprotein of Ebola viruses.

Positions 83 and 545 in the Reston virus (RESTV) glycoprotein structure (top panel). The hydrophobicity of wild-type and A83V mutant RESTV, and wild-type Ebola virus (EBOV) are shown. In the top panel, only an A83 residue on the left-most side is exposed on the surface. Residue 82 in the other two panels are only visible because they are presented with a transparent surface. A total of 50 homology models were generated using Discovery Studio 4.1 from BIOVIA (Accelrys Inc., San Diego, CA, USA). The best model was selected using probability density function total energy and discrete optimized protein energy score.

Fig. S3. Electrostatic potential of the glycoprotein of Ebola viruses.

Positions 83 and 545 in the RESTV GP structure (top panel). The electrostatic potential

of wild-type and A83V mutant RESTV GP, and wild-type EBOV GP are shown. In the top panel, only an A83 residue at the left-most side is exposed on the surface. Residue 82 in the other two are only visible because they are presented with a transparent surface.

Fig. S4. Comparisons of the glycoprotein surface among wild-type Ebola virus isolate Mayinga, wild-type, and A83V mutant Reston virus.

(a) EBOV GP structure with molecular surface and ribbon representation. The same colours are used for the receptor-binding domain (RBD) and residues 82 and 544 as in Fig. 1B. The number in parentheses indicates a residue that is not exposed on the protein surface. (b) Hydrophobicity. (c) Electrostatic potential. Yellow-boxed areas are enlarged on the right. The amino acid positions (79, 80, and 83) of the Niemann-Pick C1 (NPC1) RBD are based on the EBOV GP [19]. The actual positions of these EBOV residues in RESTV GP are 80, 81, and 84.

Fig. S5. Bar graph of mutation energy using the structure of human Niemann-Pick C1.

The bar is coloured white or black when mutation energy is significant (less than 0.5 means "stabilizing", whereas more than 0.5 means "destabilizing"). All mutations indicate amino acid variations in mammalian host species.

Fig. S6. Hydrophobicity and electrostatic potential of the surface of Niemann-Pick

C1.

Hydrophobicity (left) and electrostatic potential (right) of the human NPC1 C domain structure are shown. Residues that have many variations in non-human host species are highlighted with dotted lines.

Fig. S1

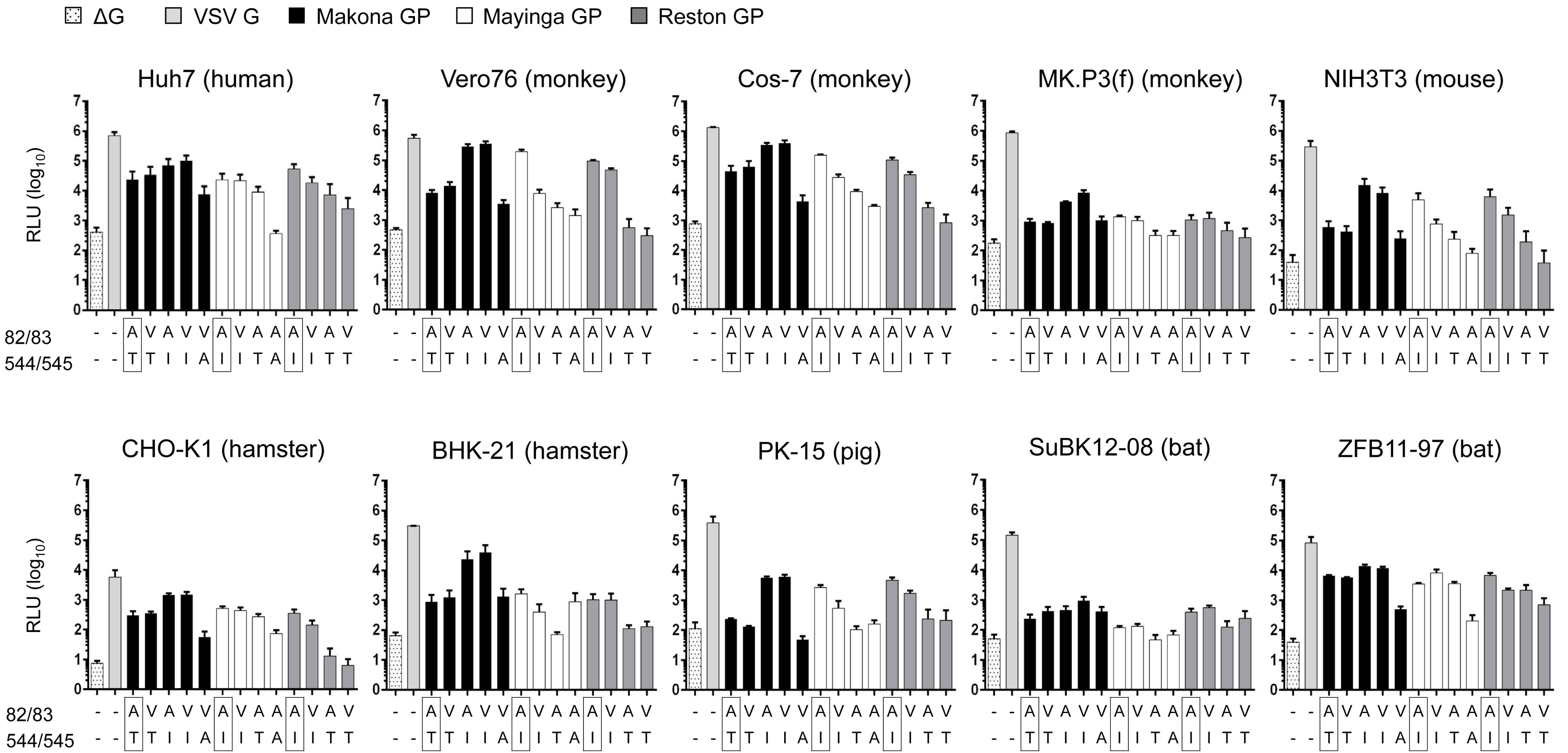

ΔG
VSV G
Makona GP
Mayinga GP
Reston GP
Huh7 (human)
Vero76 (monkey)
Cos-7 (monkey)
MK.P3(f) (monkey)
NIH3T3 (mouse)
CHO-K1 (hamster)
BHK-21 (hamster)
PK-15 (pig)
SuBK12-08 (bat)
ZFB11-97 (bat)
RLU (log10)
82/83
- - A V A V V A V A A A V A V
544/545
- - T T I I A I I T A I I T T

Fig. S2

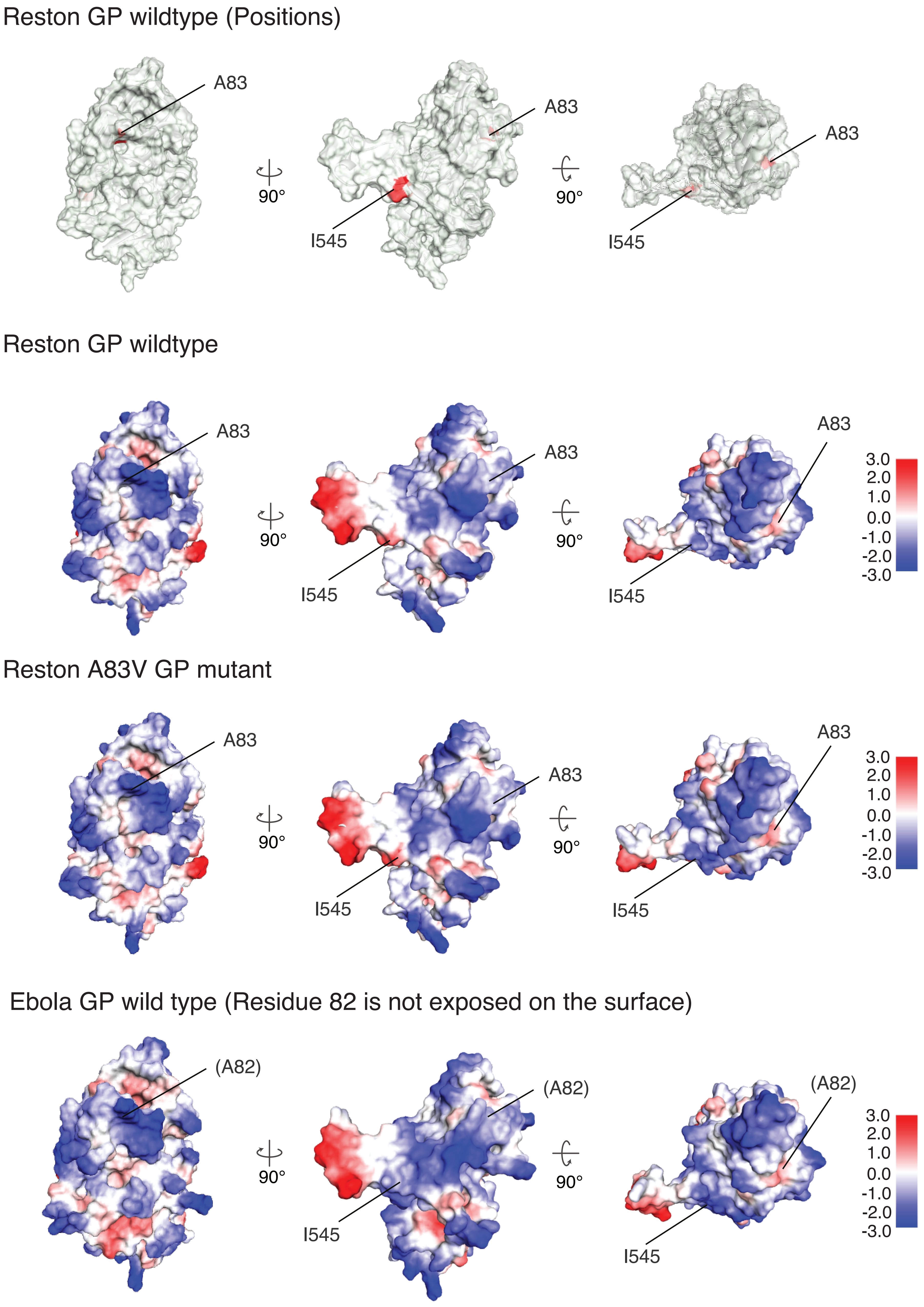

Reston GP wildtype (Positions)
A83
90°
A83
I545
90°
A83
I545
Reston GP wildtype
A83
90°
A83
I545
90°
A83
I545
3.0
2.0
1.0
0.0
-1.0
-2.0
-3.0
Reston A83V GP mutant
A83
90°
A83
I545
90°
A83
I545
3.0
2.0
1.0
0.0
-1.0
-2.0
-3.0
Ebola GP wild type (Residue 82 is not exposed on the surface)
(A82)
90°
(A82)
I545
90°
(A82)
I545
3.0
2.0
1.0
0.0
-1.0
-2.0
-3.0

Fig. S3

Reston GP wildtype (Positions)

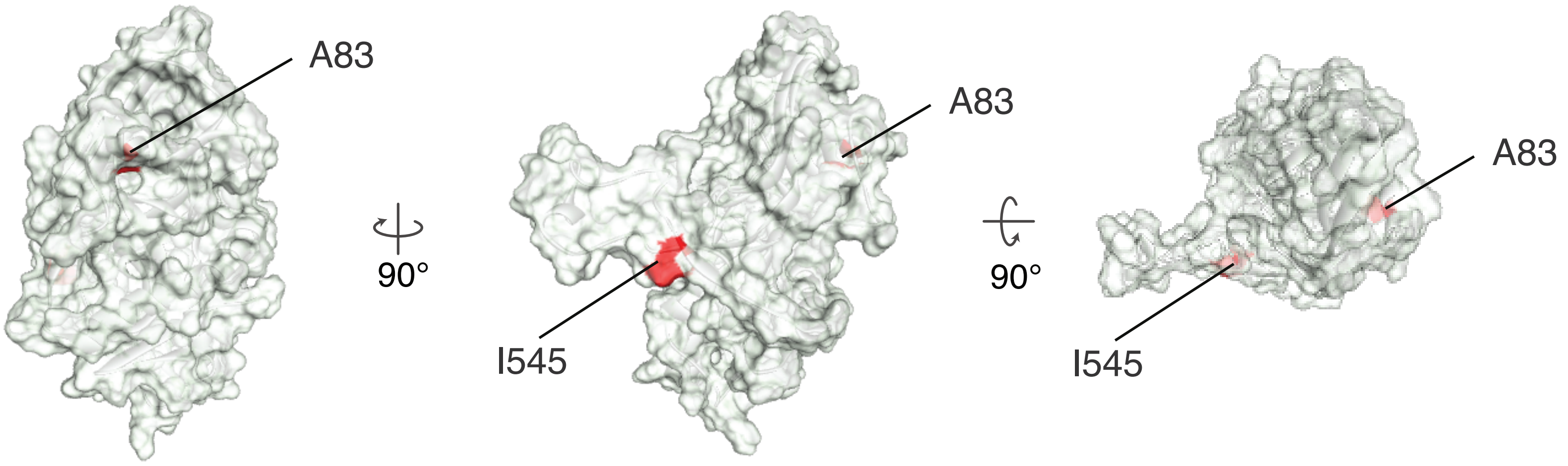

Reston GP wildtype

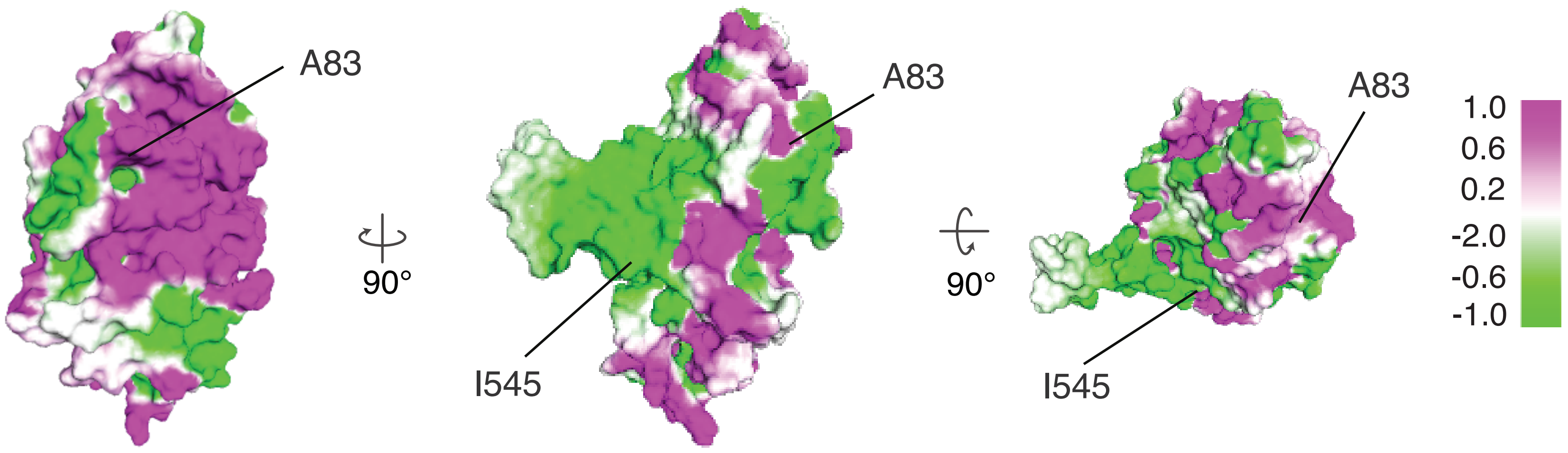

Reston A83V GP mutant

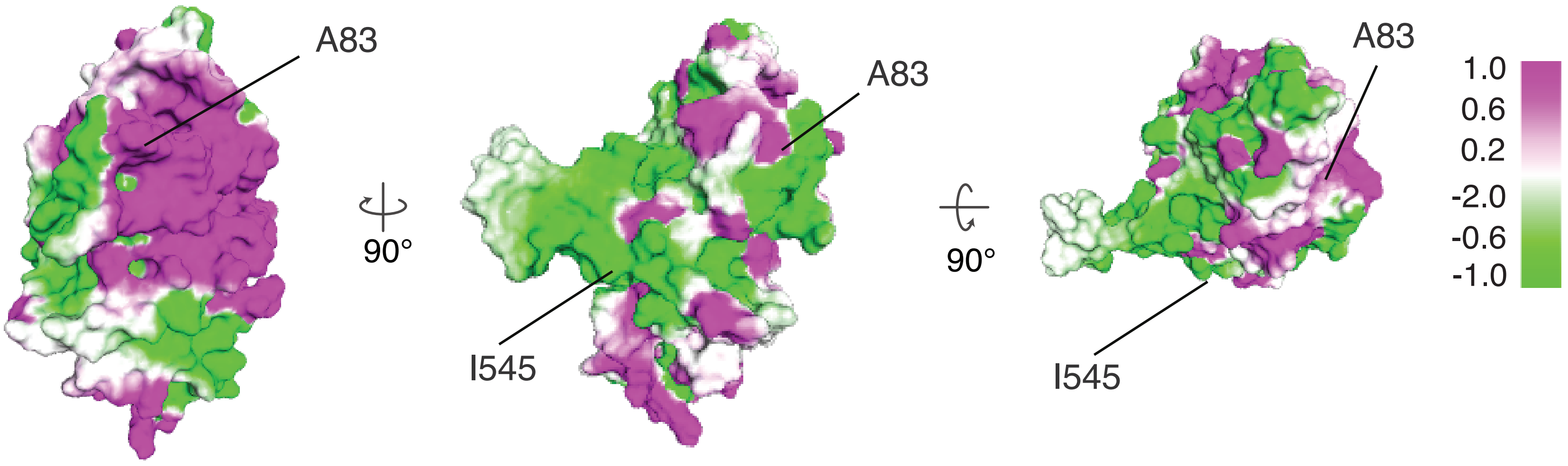

Ebola GP wild type (Residue 82 is not exposed on the surface)

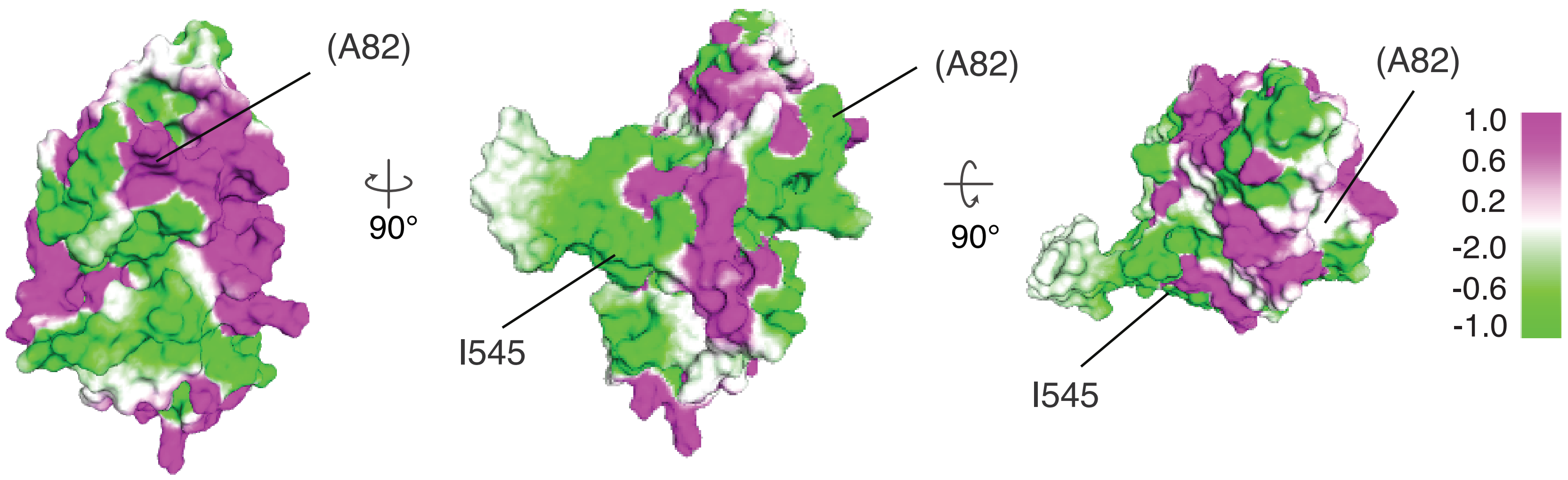

Fig. S4

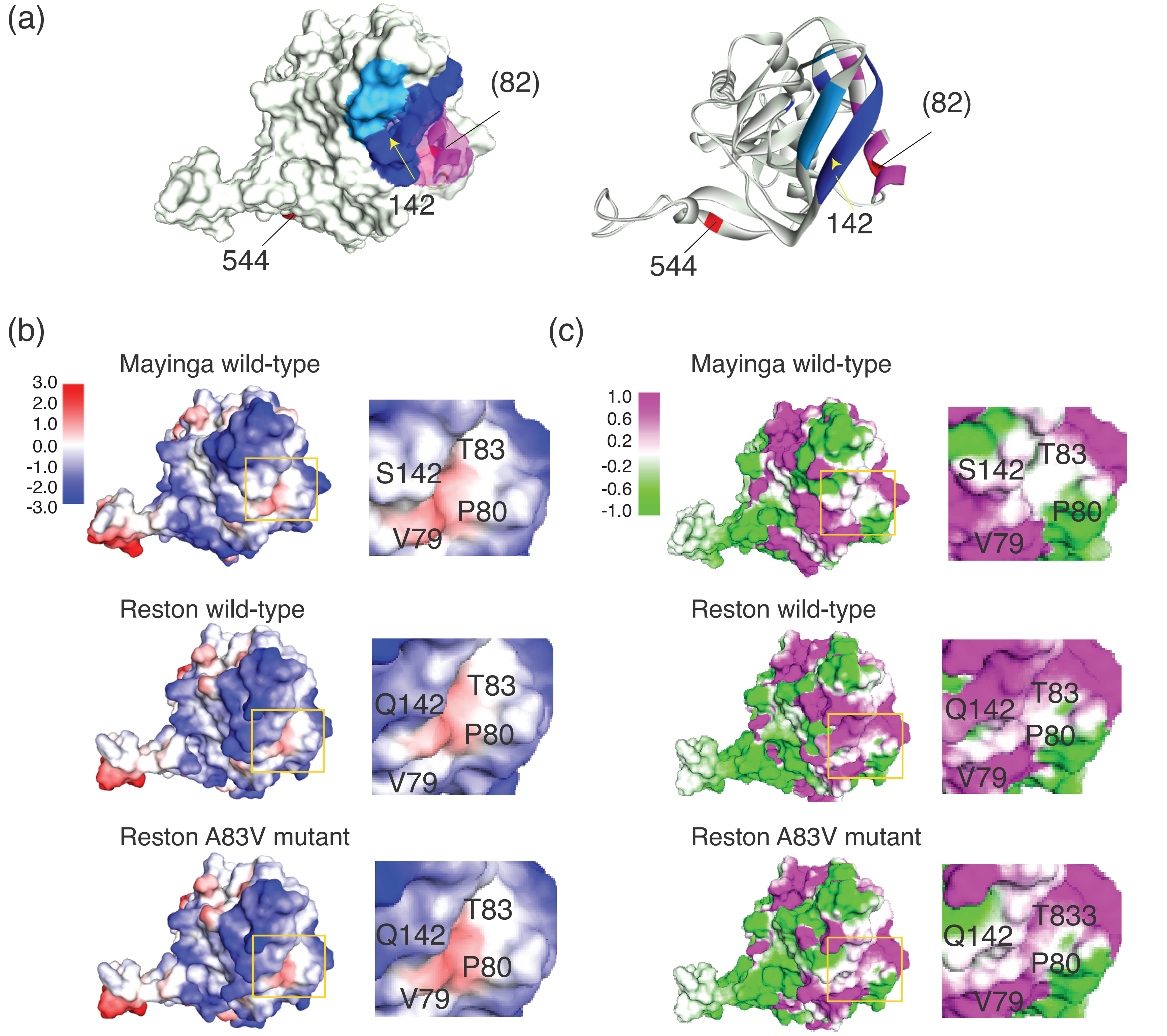
(a)
(82)
142
544
(82)
142
544
(b)
Mayinga wild-type
3.0
2.0
1.0
0.0
-1.0
-2.0
-3.0
T83
S142
P80
V79
Reston wild-type
T83
Q142
P80
V79
Reston A83V mutant
T83
Q142
P80
V79
(c)
Mayinga wild-type
1.0
0.6
0.2
-0.2
-0.6
-1.0
S142
T83
P80
V79
Reston wild-type
T83
Q142
P80
V79
Reston A83V mutant
T833
Q142
P80
V79

Fig. S5

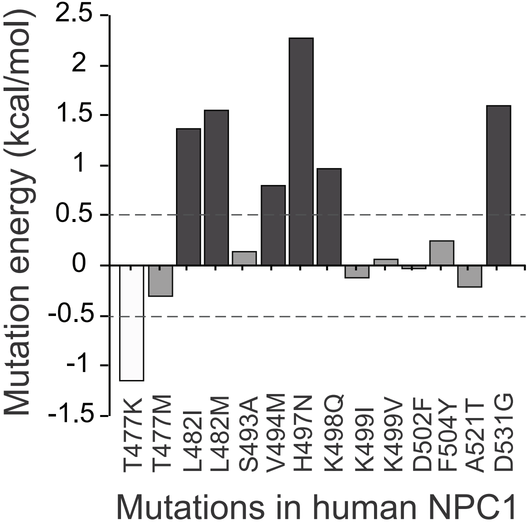

Mutation energy (kcal/mol)
2.5
2
1.5
1
0.5
0
-0.5
-1
-1.5
T477K
T477M
L482I
L482M
S493A
V494M
H497N
K498Q
K499I
K499V
D502F
F504Y
A521T
D531G
Mutations in human NPC1

Fig. S6

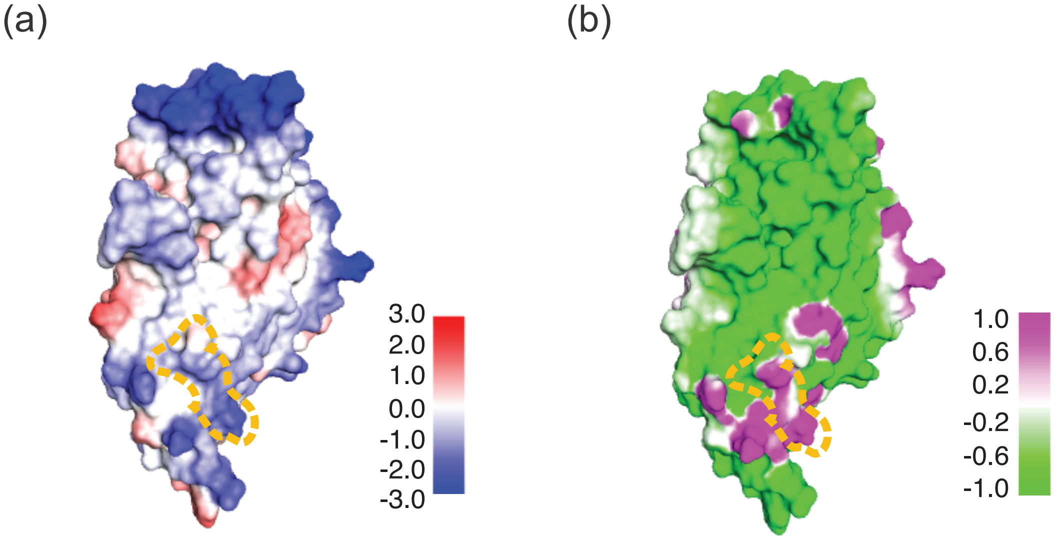

Supplement: Supplementary File 1 [file jgv-99-181-s001.pdf]
